# Evolution of psychopathology, purpose in life, and moral courage in healthcare workers during the COVID-19 pandemic: a longitudinal study

**DOI:** 10.3389/fpubh.2023.1259001

**Published:** 2023-11-08

**Authors:** Iván Echeverria, Lorena Roselló-Jiménez, Ana Benito, Luz Angela Rojas-Bernal, Marcelo O’Higgins, Gonzalo Haro

**Affiliations:** ^1^TXP Research Group, Universidad Cardenal Herrera-CEU, CEU Universities, Castellón de la Plana, Spain; ^2^Department of Mental Health, Consorcio Hospitalario Provincial de Castellón, Castellón de la Plana, Spain; ^3^Department of Basic Psychology, Clinic and Psychobiology, Jaume I University, Castellón de la Plana, Spain; ^4^Torrente Mental Health Unit, Hospital General Universitario de Valencia, Torrente, Spain; ^5^Instituto del cerebro, Hospital Universitario Mayor Méderi-Universidad del Rosario, Bogotá, Colombia; ^6^Department of Psychiatry, School of Medical Sciences, National University of Asunción, Asunción, Paraguay

**Keywords:** anxiety, burnout, COVID-19, depression, longitudinal, moral courage, post-traumatic stress disorder, purpose in life

## Abstract

**Introduction:**

Almost 2 years and five infection waves after the COVID-19 pandemic started, healthcare workers continued dealing with the pandemic situation and facing the health consequences and the mental health disorders it caused. This study aimed to evaluate the onset and progression of psychopathology as well as the role of predictor variables such as purpose in life and moral courage among healthcare workers during this time.

**Materials and methods:**

This was a longitudinal prospective study carried out with 45 Spanish healthcare workers who answered two questionnaires, the first questionnaire in April–May 2020 (T1) and the second questionnaire in September–October 2021 (T2).

**Results:**

Although 29.5% of the sample considered that their mental health had improved over this time, almost half of them (47.7%) said it had not changed, while 22.7% reported a decline in their mental health from the first time they were asked. Specifically, 46.8% presented anxiety, 23.4% depression, and 42.6% acute stress at T1, and 38.3% had anxiety, 17% depression, and 27.7% post-traumatic stress disorder at T2. Despite this, there were no differences between T1 and T2 anxiety scores (*p* = 0.53), although there was a decrease in depression (*p* = 0.03) and acute stress (*p* = 0.02) scores. Predictor variable outcomes such as purpose in life (*p* = 0.88) and moral courage (*p* = 0.86; *p* = 0.38) did not change over time, but when modelling the data, purpose in life predicted psychopathology at T1, which in turn affected the psychopathology results at T2.

**Conclusion:**

This study showed that, although psychopathology decreased over the months, its prevalence remained high. Even though the purpose in life predicted psychopathology at T1, it seems that once the psychopathology is established (T2), the factors that would improve it would be different from the protective factors that prevented its establishment, which become secondary.

## 1. Introduction

In late 2019, a new coronavirus variant called SARS-CoV-2, responsible for COVID-19 disease, was first detected in Wuhan (China); it changed societal behaviour and soon overtook health systems worldwide. One of the most affected populations was healthcare workers (HCWs), who had to face both work and personal COVID-19-related difficulties (1).

At the beginning of the pandemic, HCWs had to deal with exposure to an unknown virus, a high infection rate, and a lack of the required personal protective equipment (2, 3). They also faced staffing shortages, which in many cases resulted in overwhelming workloads and increased working hours (4). As a result, HCWs worldwide have been prone to developing psychopathology and burnout (5, 6), a dysfunctional response to prolonged work stress characterised by the appearance of emotional exhaustion, depersonalisation, and low personal fulfillment (7) that has also been associated with the development of psychopathology (8). In this regard, coping styles may have played a relevant role in the burnout, and thus the psychopathology of HCWs during COVID-19, as avoidance-oriented and maladaptive coping predicted burnout (9). One of the most affected countries during the early phases of the COVID-19 pandemic was Spain, where HCWs showed high rates of anxiety, depression, post-traumatic stress disorder (PTSD), and medium and high levels of burnout (7, 10). In addition to their work, HCWs also had to cope with personal and family concerns such as social isolation, managing the work-life balance, and the risk of transmitting the virus to their loved ones. In fact, a previous study demonstrated that the latter would be the main reason why HCWs would not go to work (11), and one of the concerns that has been responsible for the negative impact that the pandemic has had on their mental health, resulting in a high prevalence of anxiety, depression, and acute stress (12).

In the fifth wave, the situation changed: the initial shortage of personnel and resources improved, the workload decreased, new information on SARS-CoV-2 became available, and society recovered most of its usual activities (13, 14). However, several longitudinal studies from remarkably different countries, such as Singapore, Germany, or Australia, have shown long-term psychopathology and burnout (15–17). COVID-19 and long COVID, a condition involving persistent long-term symptoms of SARS-CoV-2 infection, have also been associated with neuropsychiatric symptoms such as anxiety or depression (18). In this regard, there are precedents for long-lasting mental health problems among HCWs due to epidemics. For example, 1 year after the SARS outbreak, HCWs with high-risk exposure to SARS during the outbreak continue to show higher levels of perceived stress than those with low-risk exposure. This perceived stress was associated with high levels of anxiety, depression, and post-traumatic stress scores (19).

The causes of the onset, evolution, and maintenance of psychopathology are varied and its mechanisms are complicated. However, some dimensions related to the characteristics of HCWs may have had an influence, including purpose in life (PIL) and moral courage (MC). PIL refers to the perception an individual has about the purpose and value of their life (12). Several studies prior to the COVID-19 pandemic have demonstrated the predictive role of PIL in the development of psychopathology (20, 21), and during the pandemic, high levels of PIL were associated with a lower prevalence of psychopathology in HCWs (12). MC is defined as the ability to face danger or social disapproval when performing what one believes to be their duty (22). Paradoxically, not being able to act in accordance with these moral values may generate “moral distress” and, in turn, favour the onset of psychopathology (23). The role of MC and moral distress may have been especially important in the early stages of the COVID-19 pandemic when, because of the lack of resources, HCW had to prioritise which patients received treatment or even decide to risk their own health to help patients (24).

Although several studies have registered the prevalence of mental disorders such as anxiety, depression, PTSD, and burnout during epidemics and pandemics, none have related the longitudinal evolution of psychopathology as a function of factors such as PIL or MC. Therefore, in this current study, we aimed to explore the evolution of the mental health status of HCWs throughout the pandemic in terms of these predictors and provide a broader perspective on this issue. We hypothesised that (1) psychopathology would decrease in HCWs over time; (2) the scores for PIL and MC at T1 would predict the psychopathology and burnout levels measured in the HCWs at T2.

## 2. Materials and methods

Given the research objective, we designed an observational prospective study. A Spanish cohort of 47 HCWs (including physicians, nurses, nursing assistants, administrative staff, etc.) was recruited by convenience sampling from the Consorcio Hospitalario Provincial de Castellón (Spain), the second largest hospital in the city. The G*Power software (v3.1.9.4) was used to calculate that, considering an expected effect size of *d* = 0.2, an alpha of 5%, and a beta of 5%, the critical sample size was 41 and a total sample size of 67 would be required when performing the sign test.

The first assessment was obtained from a previous study (12) that evaluated the sample in April–May 2020 (T1), during the peak of the first wave of COVID-19 in Spain. The second assessment was completed by the same sample in September–October 2021 (T2), just after the fifth wave of COVID-19.

After signing their informed consent, the study participants completed a series of self-administered questionnaire-based instruments in Spanish. All these instruments have been previously validated for Spanish speakers and have already been used in the COVID-19 research context (7, 12, 22). The questionnaires could be completed online or by hand. In T1, we distributed both the online and handwritten versions in each hospital department. Participants were asked for permission to be contacted again after a period of time. In T2, we re-contacted participants by email and sent them the online version, as well as providing the handwritten version in the same hospital departments for those who preferred this option.

First, they completed a sociodemographic questionnaire that asked about their age, sex, marital status, religiosity, professional category, role of responsibility, history of physical conditions or mental health disorders, and whether they smoked.

As independent and predictor variables, personal and family/friends’ exposure to SARS-CoV-2 was assessed using a questionnaire for this purpose (12); PIL was analysed using the PIL scale (25), and a dichotomous variable was also calculated to differentiate between individuals who had a sense of PIL and those who did not [cutoff point (CP) = 113]; and MC was assessed with the Moral Courage Scale for Physicians (MCSP) (26) and the Professional Moral Courage Scale (PMCS) (27).

Variables that evaluated psychopathology and burnout were considered dependent variables. Total scores and dichotomous variables for these were calculated, and the participants were classified into individuals that exceeded the CP of each scale and those that did not. Anxiety was assessed using the Beck Anxiety Inventory (28) (BAI; CP = 8), depression using the Beck Depression Inventory (29) (BDI-II; CP = 14), acute stress disorder using the Acute/Post-Traumatic Stress Disorder Scale (12) (ETEA-PT; CP = 9), and PTSD at T2 by considering the additional ETEA-PT questionnaire item that asks if the symptoms lasted more than 1 month. Drug abuse was assessed with the Drug Abuse Screening Test-10 (30) (DAST-10; CP = 1) and alcohol abuse was tested employing the Alcohol Use Disorders Identification Test (31) (AUDIT; CP for women = 6, CP for men = 8). The Maslach Burnout Inventory-Human Services Survey (MBI-HSS) (32) was used to evaluate the presence of burnout at T2, defining high levels either of emotional exhaustion (CP ≥27) or depersonalisation (CP ≥10) (33).

The SPSS software (version 27) for Microsoft (IBM Corp., Armonk, NY), a reliable and valid data analysis tool (34), was used for all the statistical analyses. After the exploratory (normality, independence, homoscedasticity, linearity, and non-collinearity) and descriptive studies, the variables were compared using the sign test for quantitative variables and the Pearson chi-square test for categorical variables. Generalized linear models and logistic regressions were created for the dependent variables, introducing personal and family/friends’ exposure to SARS-CoV-2, PIL, PMCS, and psychopathology scale scores at T1. MCSP was excluded from the regression analyses due to collinearity problems with PMCS, as both measure variables were related to MC and therefore significantly correlated (*r* = 0.417; *p* = 0.007). Finally, the data were modelled using the PROCESS plugin (v3.4) for SPSS, a well-known tool for this purpose (35). The use of these programmes is supported by current studies (7, 12, 22).

The ethical principles set out in the Declaration of Helsinki and by the Council of Europe Convention were followed, and the informed consent of all participants was obtained. Moreover, data confidentiality was guaranteed according to the General Data Protection Regulation (GDPR; 2018). This study was authorised by the Institutional Review Board of the Consorcio Hospitalario Provincial de Castellón (ref. A-15/04/20) and the Clinical Research Ethics Committee at the Cardenal Herrera-CEU University (ref. CEI20/068).

## 3. Results

### 3.1. Sociodemographic characteristics

Table 1 shows the sociodemographic characteristics of the sample.

**Table 1:** Sociodemographic characteristics of the study sample.

	*n* = 45 % (*n*)/*M* (SD)
Age	43.8 (11.8)
Sex (female)	70.2 (33)
*Marital status*	
Married	59.6 (28)
Single	27.7 (13)
Divorced	10.6 (5)
Widowed	2.1 (1)
Religiosity (yes)	53.2 (25)
*Professional category*	
Physician	34 (16)
Nurse	31.9 (15)
Nursing assistant	12.8 (6)
Administrative staff	6.4 (3)
Psychologist	2.4 (3)
Ancillary nurse	2.1 (1)
Pharmacist	2.1 (1)
Security staff	2.1 (1)
Occupational therapist	2.1 (1)
Social worker	2.1 (1)
Role of responsibility	17 (8)
History of a physical condition	28.3 (13)
History of a mental health disorder	21.3 (11)
Smoker	23.9 (11)

*n*, number of participants; %, percentage; *M*, mean; SD, standard deviation.

Of the total of 47 Spanish HCWs evaluated, the majority were women (70.2%; *n* = 33), and the mean (*M*) age was 43.8 years. Almost 60% of the sample were married (59.6%; *n* = 28), and around half reported being practicing Christians, i.e., religious (53.2%; *n* = 25). Regarding their professional category, the sample mostly comprised physicians (34%; *n* = 16), nurses (31.9%; *n* = 15), and nursing assistants (12.8%; *n* = 6), followed by administrative staff (6.4%, n = 3). Of these, 17% (*n* = 8) held positions of responsibility. In terms of their health, 28.3% (*n* = 13) suffered from a physical condition, 21.3% (*n* = 11) had a history of having suffered from a mental disorder, and 23.9% (*n* = 11) were smokers.

No significant differences in sociodemographic characteristics were observed between T1 and T2.

### 3.2. Evolution of SARS-CoV-2 exposure, purpose in life, and moral courage

Table 2 shows the evolution of personal and family/friends’ exposure to SARS-CoV-2, PIL, and MC at T1 and T2.

**Table 2:** COVID-19 exposure, purpose in life, moral courage, psychopathology, and burnout at T1 and T2.

	T1 *n* = 45% (*n*)/Me (IQR)	T2 *n* = 45% (*n*)/Me (IQR)	*p*-value
Personal and family/friends’ exposure to SARS-CoV-2	0.5 (2)	1 (2)	0.07
PIL (score)	111 (19)	110 (21)	0.88
PIL (yes)	53.2 (25)	55.3 (26)	
MCSP	8 (2)	8 (2)	0.38
PMCS	11 (2)	11 (1.25)	0.86
BAI	5.5 (12.25)	5 (10)	0.53
Anxiety (yes)	47.8 (22)	38.3 (18)	
BDI-II	7 (11)	4 (11)	0.03^*^
Depression (yes)	23.4 (11)	17 (8)	
ETEA-PT	6 (9)	4 (8)	0.02^*^
Acute stress (yes)	42.6 (20)	27.7 (13)	
PTSD (yes)		27.7 (13)	
DAST-10	0 (0)	0 (0)	0.62
Drug (yes)	6.4 (3)	6.4 (3)	
AUDIT	2.5 (2.1)	2.5 (3)	1
Alcohol (yes)	10.6 (5)	6.4 (3)	
Psychopathology (score)	20 (31)	18 (26)	0.02^*^
Al least one mental disorder (yes)	61.7 (29)	53.2 (25)	
MBI-HSS		−25 (44)	
Burnout (yes)		34 (16)	

*n*, number of participants; %, percentage; Me, median; IQR, interquartile range; T1, first assessment; T2, second assessment; PIL, purpose in life; MCSP, Moral Courage Scale for Physicians; PMCS, Professional Moral Courage Scale; BAI, Beck Anxiety Inventory; BDI-II, Beck Depression Inventory; ETEA-PT, Acute/Post-Traumatic Stress Disorder Scale; PTSD, Post-Traumatic Stress Disorder; DAST-10, Drug Abuse Screening Test-10; AUDIT, Alcohol Use Disorders Identification Test; MBI-HSS, Maslach Burnout Inventory-Human Services Survey. ^*^p < 0.05.

#### 3.2.1. Personal and family/friends’ exposure to SARS-CoV-2

HCWs reported lower personal and family/friends’ exposure to SARS-CoV-2 at T1 [Median (Me) = 0.5; interquartile range (IQR) = 2] than at T2 (Me = 1; IQR = 2), although this finding did not reach significance (*p* = 0.07).

#### 3.2.2. Purpose in life and moral courage

Some 53.2% (*n* = 25) of the sample presented low PIL at T1 and 55.3% (*n* = 26) showed low PIL at T2. Thus, 42.6% (*n* = 20) presented low PIL at both T1 and T2, and 10.6% (*n* = 5) presented low PIL at T1 but not at T2. In turn, 34% (*n* = 16) of the sample showed high PIL at both T1 and T2.- No significant differences were found between T1 and T2 PIL scores (Me = 111, IQR = 19 vs. Me = 110, IQR = 21; *p* = 0.88).

No significant differences were found between T1 and T2 MCSP (Me = 8, IQR = 2 vs. Me = 8, IQR = 2; *p* = 0.38) or PMCS scores (Me = 11, IQR = 2 vs. Me = 11, IQR = 1.25; *p* = 0.86).

### 3.3. Evolution of self-perceived mental health, psychopathology, and burnout

#### 3.3.1. Self-perceived mental health and psychopathology

Table 2 shows the evolution of psychopathology at T1 and T2.

Almost half of the sample (47.7%; *n* = 21) said there had been no changes in their mental health since they were first asked at T1, while 22.7% (*n* = 10) reported a decline. Lastly, 29.5% (*n* = 13) considered that their mental health had improved over time. When stratifying these results to those with psychopathology only at T2 or both T1 and T2, 40.9% (*n* = 9) said there had been no changes, 40.9% (*n* = 9) cited a decline, and 18.2% (*n* = 4) reported an improvement.

In turn, 47.8% (*n* = 22) of the sample presented anxiety at T1, while 38.3% (*n* = 18) reported it at T2. Thus, 28.3% (*n* = 13) presented anxiety at both T1 and T2, and 19.5% (*n* = 9) that had presented anxiety at T1 did not report it at T2. Nonetheless, most of the sample (43.5%; *n* = 20) said they had not experienced anxiety in either T1 or T2. Furthermore, no significant differences were found between T1 and T2 BAI scores (Me = 5.5, IQR = 12.25 vs. Me = 5, IQR = 10; *p* = 0.53).

Regarding depression, 23.4% (*n* = 11) of the sample presented it at T1 and 17% (*n* = 8) at T2. Thus, 14.9% (*n* = 7) had remained depressed at both T1 and T2, 8.5% (*n* = 4) that had presented depression in T1 did not report at T2, and 74.5% (*n* = 35) said they were not depressed at either time point. Nevertheless, the BDI-II scores were significantly decreased from T1 to T2 (Me = 7, IQR = 11 vs. Me = 4; IQR = 11; *p* = 0.03).

Some 42.6% (*n* = 20) of the sample presented acute stress at T1, and 27.7% (*n* = 13) reported it at T2. Thus, 19.1% (*n* = 9) of the sample presented it at both T1 and T2, and 8.5% (*n* = 4) who did not have acute stress at T1 showed it at T2. Therefore, more than a quarter of the sample (27.7%; *n* = 13) stated at T2 that they had had acute stress for more than a month, meaning that they had developed PTSD. Also of note is that 48.9% (*n* = 23) of the sample did not show acute stress at any time and that 23.4% (*n* = 11) with acute stress at T1 did not present it at T2. In fact, scores in the ETEA-PT decreased from T1 to T2 (Me = 6, IQR = 9 vs. Me = 4, IQR = 8; *p* = 0.02).

In terms of the use of drugs, 6.4% (*n* = 3) reported having done so at T1, and 6.4% (*n* = 3) reported at T2. Thus, 4.3% (*n* = 2) said they used drugs at both T1 and T2. However, most of the sample did not report drug abuse (97.7%; *n* = 43) in either T1 or T2. Indeed, there were no significant differences in the DAST-10 scores between T1 and T2 (Me = 0, IQR = 0 vs. Me = 0, IQR = 0; *p* = 0.62).

Regarding alcohol abuse, there was 10.6% (*n* = 5) of abuse reported at T1 and 6.4% (*n* = 3) at T2. Thus, 4.3% (*n* = 2) presented this problem at both T1 and T2, although most of the sample did not report alcohol abuse (93%; *n* = 40) in either T1 or T2. Moreover, there were no significant differences in the AUDIT scores between T1 and T2 (Me = 2.5, IQR = 2.1 vs. Me = 2.5, IQR = 3; *p* = 1).

Considering all the aforementioned, 61.7% (*n* = 29) had at least one mental disorder at T1, while 53.2% (*n* = 25) at T2. Similarly, higher overall psychopathology scores were reported at T1 than at T2 (Me = 20, IQR = 31 vs. Me = 18, IQR = 26; *p* = 0.02).

#### 3.3.2. Burnout

Finally, in reference to burnout, at T2, 17% (*n* = 8) showed high scores in emotional exhaustion (Me = 12, IQR = 18), 27.7% (*n* = 13) had high scores in depersonalisation (Me = 5, IQR = 9), and 23.4% (*n* = 11) presented low scores in personal accomplishment (Me = 41, IQR = 11) subscales. Thus, 34% (*n* = 16) reached the CP for the depersonalisation or emotional exhaustion subscales, which was the criterion to be considered as having burnout (Table 2).

### 3.4. Generalized linear models, logistic regressions, and psychopathology data models

Table 3 shows the generalized linear models predicting psychopathology at T1.

**Table 3:** Generalized linear models predicting psychopathology at T1.

Response	Predictors^a^	OR (95% confidence interval) *p*-value
BAI	PIL (T1)	0.67 (0.56, 0.81); *p* < 0.001^***^
	Personal and family/friends’ exposure to SARS-CoV-2 (T1)	8.01 (2.17, 29.53); *p* = 0.002^**^
BDI-II	PIL (T1)	0.67 (0.57, 0.79); *p* < 0.001^***^
	Personal and family/friends’ exposure to SARS-CoV-2 (T1)	7.67 (1.27, 46.32); *p* = 0.02^*^
ETEA-PT	PIL (T1)	0.72 (0.64, 0.80); *p* < 0.001^***^
	Personal and family/friends’ exposure to SARS-CoV-2 (T1)	5.76 (2.14, 15.53); *p* = 0.001^**^
Psychopathology	PIL (T1)	0.30 (0.18, 0.49); *p* < 0.001^***^
	Personal and family/friends’ exposure to SARS-CoV-2 (T1)	328.84 (8.87, 12,184.10); *p* = 0.002^**^

OR, odds ratio; PIL, purpose in life; BAI, Beck Anxiety Inventory; BDI-II, Beck Depression Inventory; ETEA-PT, Acute/Post-Traumatic Stress Disorder Scale; DAST-10, Drug Abuse Screening Test-10; AUDIT, Alcohol Use Disorders Identification Test. ^*^p < 0.05, ^**^p < 0.01, and ^***^p < 0.001.

a Personal and family/friends’ exposure to SARS-CoV-2, PMCS, and PIL scores at T1 were introduced as predictor variables.

T1 PIL scores predicted T1 BAI [OR = 0.67; 95% CI (0.56, 0.81); *p* < 0.001], BDI-II [OR = 0.67; 95% CI (0.57, 0.79); *p* < 0.001], and ETEA-PT scores [OR = 0.72; 95% CI (0.64, 0.80); *p* < 0.001]. Thus, it also predicted T1 overall psychopathology scores [OR = 0.30; 95% CI (0.18, 0.49); *p* < 0.001]. On the other hand, T1 personal and family/friends’ exposure to SARS-CoV-2 scores predicted T1 BAI [OR = 8.01; 95% CI (2.17, 29.53); *p* = 0.002], BDI-II [OR = 7.67; 95% CI (1.27, 46.32); *p* = 0.02], and ETEA-PT scores [OR = 5.76; 95% CI (2.14, 15.53); *p* = 0.001]. Thus, it also predicted T1 overall psychopathology scores [OR = 328.84; 95% CI (8.87, 12,184.10); *p* = 0.002].

Table 4 shows the generalized linear models and logistic regression predicting psychopathology and burnout at T2.

**Table 4:** Generalized linear models and logistic regression predicting psychopathology and burnout at T2.

Response	Predictors^a^	OR (95% confidence interval) *p*-value	Predictors^b^	OR (95% confidence interval) *p*-value
BAI	PIL (T1)	0.68	BAI (T1)	1.90
				(1.46, 2.49)
		(0.54, 0.85)		
				*p* < 0.001^***^
		*p* = 0.001^**^		
BDI-II	PIL (T1)	0.72	BDI-II (T1)	1.88
		(0.59, 0.87)		(1.24, 2.85)
				*p* = 0.003^**^
		*p* = 0.001^**^		
ETEA-PT	PIL (T1)	0.80	ETEA-PT (T1)	1.36
				(1.01, 1.83)
		(0.70, 0.91)		
		*p* = 0.001^**^		*p* = 0.04^*^
PTSD	—	—	ETEA-PT (T1)	1.14
				(1.02, 1.27)
				*p* = 0.01^*^
DAST-10	—	—	DAST-10 (T1)	2.98
				(2.44, 3.64)
				*p* < 0.001^***^
AUDIT	—	—	AUDIT (T1)	1.70
				(1.40, 2.06)
				*p* < 0.001^***^
Psychopathology	PIL (T1)	0.38	Psychopathology (T1)	1.84
		(0.23, 0.62)		(1.33, 2.54)
		*p* < 0.001^***^		
				*p* < 0.001^***^
MBI-HSS	PIL (T1)	0.47	—	—
		(0.32, 0.70)		
		*p* < 0.001^***^		
MBI-HSS—emotional exhaustion	PIL (T1)	0.69	—	—
		(0.56, 0.86)		
		*p* = 0.001^**^		
MBI-HSS—depersonalisation	PIL (T1)	0.86	—	—
		(0.76, 0.96)		
		*p* = 0.01^*^		
MBI-HSS—personal accomplishment	PIL (T1)	1.26	—	—
		(1.12, 1.41)		
		*p* < 0.001^***^		

OR, odds ratio; PIL, purpose in life; BAI, Beck Anxiety Inventory; BDI-II, Beck Depression Inventory; ETEA-PT, Acute/Post-Traumatic Stress Disorder Scale; PTSD, Post-Traumatic Stress Disorder; DAST-10, Drug Abuse Screening Test-10; AUDIT, Alcohol Use Disorders Identification Test; MBI-HSS, Maslach Burnout Inventory-Human Services Survey. ^*^p < 0.05, ^**^p < 0.01, and ^***^p < 0.001.

a Personal and family/friends’ exposure to SARS-CoV-2, PMCS, and PIL scores at T1 were introduced as predictor variables.

b Personal and family/friends’ exposure to SARS-CoV-2, PMCS, PIL, and overall psychopathology scores at T1 were introduced as predictor variables.

T1 PIL scores predicted T2 BAI [OR = 0.68; 95% CI (0.54, 0.85); *p* = 0.001], BDI-II [OR = 0.72; 95% CI (0.59, 0.87); *p* = 0.001], and ETEA-PT scores [OR = 0.80; 95% CI (0.70, 0.91); *p* = 0.001]. Thus, it also predicted T2 overall psychopathology scores [OR = 0.38; 95% CI (0.23, 0.62); *p* < 0.001]. In turn, T1 PIL scores predicted T2 MBI-HSS scores [OR = 0.47; 95% CI (0.32, 0.70); *p* < 0.001], emotional exhaustion [OR = 0.69; 95% CI (0.56, 0.86); *p* = 0.001], depersonalisation [OR = 0.86; 95% CI (0.76, 0.96); *p* = 0.01], and personal accomplishment [OR = 1.26; 95% CI (1.12, 1.41); *p* < 0.001] burnout subscales scores.

However, when T1 scores of each questionnaire were introduced in the regressions, T1 BAI predicted T2 BAI scores [OR = 1.90; 95% CI (1.46, 2.49); *p* < 0.001]; T1 BDI-II predicted T2 BDI-II scores [OR = 1.88; 95% CI (1.24, 2.85); *p* = 0.003]; T1 ETEA-PT predicted T2 ETEA-PT [OR = 1.36; 95% CI (1.01, 1.83); *p* = 0.04] and T2 PTSD scores [OR = 1.14; 95% CI (1.02, 1.27); *p* = 0.01]; T1 DAST-10 predicted T2 DAST-10 scores [OR = 2.98; 95% CI (2.44, 3.64); *p* < 0.001]; T1 AUDIT predicted T2 AUDIT scores [OR = 1.70; 95% CI (1.40, 2.06); *p* < 0.001]; and T1 psychopathology predicted T2 overall psychopathology scores [OR = 1.84; 95% CI (1.33, 2.54); *p* < 0.001].

**Figure 1:**
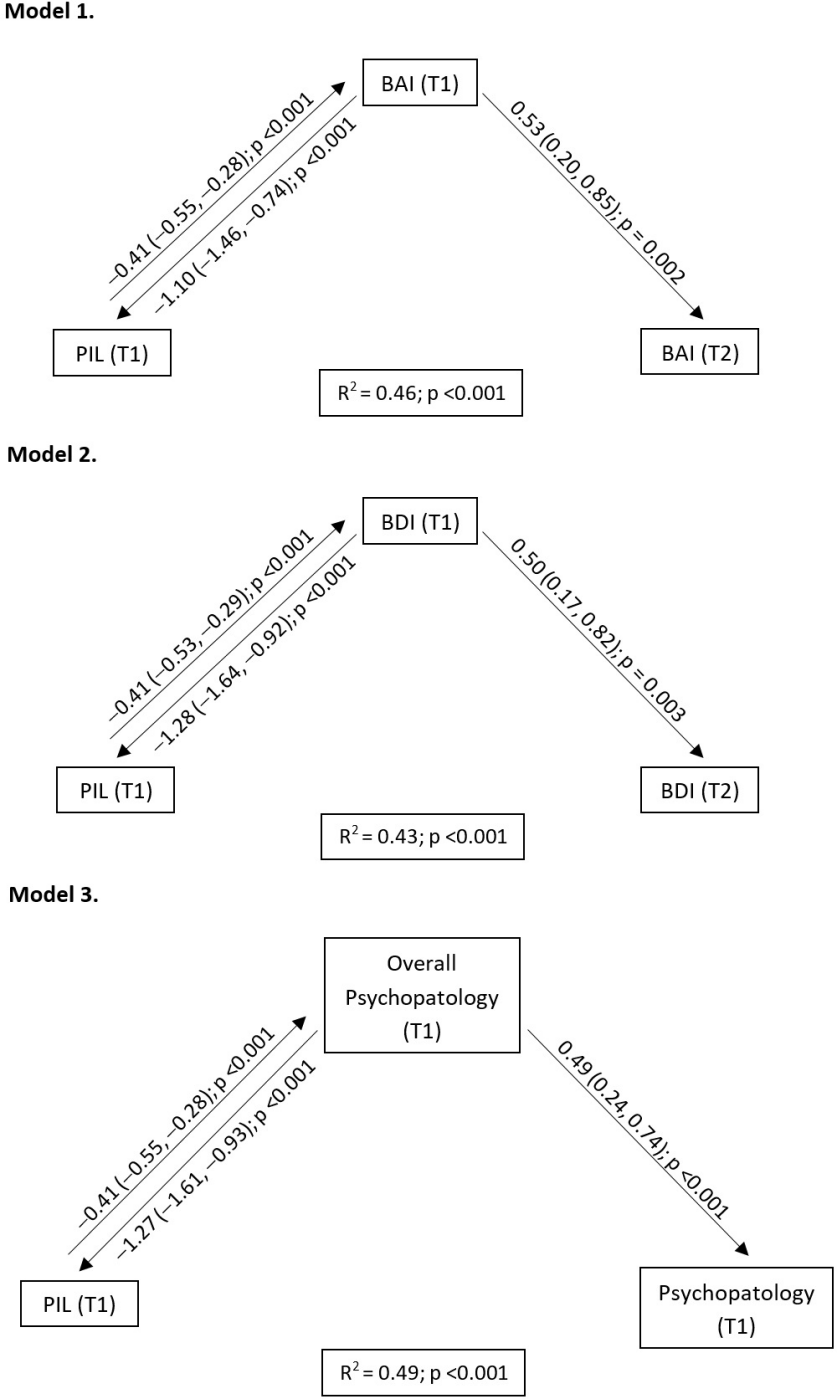
Explanatory models of the psychopathology at T1 and T2. PIL, purpose in life; BAI, Beck anxiety inventory; BDI-II, Beck depression inventory.

We modelled the data according to the results obtained in the generalized linear models, and those with best fit were included in Figure 1. Model 1 shows the reciprocal influence between T1 PIL and T1 BAI [*B* = −0.41; 95% CI (−0.55, −0.28); *p* < 0.001; *B* = −1.10; 95% CI (−1.46, −0.74); *p* < 0.001], and how T1 BAI predicted T2 BAI [*B* = 0.53; 95% CI (0.20, 0.85); *p* = 0.002]. Model 2 shows the reciprocal influence between T1 PIL and T1 BDI-II [*B* = −0.41; 95% CI (−0.53, −0.29); *p* < 0.001; *B* = −1.28; 95% CI (−1.64, −0.92); *p* < 0.001], and how T1 BDI-II predicted T2 BDI-II [*B* = 0.50; 95% CI (0.17, 0.82); *p* = 0.003]. Similarly, model 3 shows the reciprocal influence between T1 PIL and T1 psychopathology [*B* = −0.41; 95% CI (−0.55, −0.28); *p* < 0.001; *B* = −1.27; 95% CI (−1.61, −0.93); *p* < 0.001]. In addition, T1 psychopathology predicted T2 overall psychopathology scores [*B* = 0.49; 95% CI (0.24, 0.74); *p* < 0.001].

## 4. Discussion

This study aimed to longitudinally evaluate the evolution of the psychopathology presented by HCWs after the different waves of COVID-19 infections and to elucidate the role of predictors such as PIL or MC.

According to our first hypothesis, psychopathology has decreased since the beginning of the pandemic. However, this decrease is lower than would be expected, taking into account that the conditions of HCWs in the fifth wave have generally improved, with more information about the virus, personnel, resources, and vaccines available, fewer infections, a reduced workload, etc., over time. This fact coincides with the impressions given by the HCWs in the questionnaires, with most stating that their mental health had not improved (47.7%) or that it had worsened (22.7%). Nevertheless, it was striking that approximately 20% of HCWs who still had psychopathology at T2 said their mental health had improved, which is indicative of the severity of the psychopathology they initially had.

Considering the above, there may be several explanations for why there has not been a greater improvement in their mental health as time passed. The main reason may be that in the fifth wave, the pandemic was still ongoing, and even with the improving conditions, insufficient time had passed for the psychopathology to have subsided. In fact, a longitudinal study conducted in the general population (36) stated that 2 years after the onset of the COVID-19 pandemic, psychopathology scores had not yet returned to pre-pandemic scores. Moreover, a study conducted during the SARS-CoV-1 epidemic (37) showed that, 1 year after the epidemic, psychopathology prevalence was higher among HCWs than in the general population. Another research study (38) noted that further reduction in the psychopathology of HCWs may not have occurred due to the high persistence of baseline mental health disorders (in our study, more than two-thirds of those with disorders at T1 were still suffering from them at T2) and the incidence of new mental disorders during subsequent waves of COVID-19 (in our study, one in three HCWs without mental disorders at T1 developed one at T2).

Furthermore, certain elements may have worsened throughout the COVID-19 pandemic, such as personal and family/friends’ exposure to SARS-CoV-2. In that regard, this study and previous studies have shown how this type of exposure was a relevant predictor of HCWs presenting psychopathology at the onset of the pandemic (7, 12). While this exposure increased, a study conducted during the first to third wave of COVID-19 in Spain (39) showed that fear of COVID-19 contagion, which was related to the presence of anxiety and depressive symptoms (40), decreased over time. Thus, fear of contagion would be an element to take into account when assessing the role of exposure to SARS-CoV-2 in the development of psychopathology.

Although multiple studies have analysed the role of extrinsic characteristics such as those discussed above (exposure to SARS-CoV-2, availability of vaccines, work burden, etc.), few have considered intrinsic characteristics like PIL or MC in the appearance of psychopathology in HCWs during the SARS-CoV-2 pandemic. In this sense, our second hypothesis was partially fulfilled.

We found that a high PIL predicted lower anxiety, depression, and acute stress at T1 and T2, and lower burnout scores at T2, coinciding with previous cross-sectional studies conducted during the COVID-19 pandemic (7, 12, 22). Indeed, PIL is framed within the salutogenic model, which is a global orientation to perceive the world as comprehensible, manageable, and meaningful despite the stressful situations one encounters, thus acting as a coping mechanism (41). However, the predictive role of PIL on psychopathology at T2 disappeared when psychopathology at T1 was introduced, which became the only predictor of psychopathology at T2. Thus, PIL would be the main predictor that influences the onset of psychopathology but not its maintenance, where other factors that have not been studied in this work may have a relevant influence. For all of the above, MC may not have played any role.

Finally, it is important to mention the limitations of this current work. First, the main shortcoming was the lack of assessment of the occupational exposure of HCWs to SARS-CoV-2. However, by the time this study was completed, the hospital in which it was conducted had already gone through several waves of cases within the context of the COVID-19 pandemic, and so most HCWs had already been exposed. Furthermore, burnout was only assessed at T2, so burnout at T1 is unknown. However, given that burnout is by definition a dysfunctional response to prolonged work stress, the prevalence that would have been collected at such an early stage as T1 would predictably correspond to the idiosyncratic burnout of the Spanish healthcare system and not to the overload derived from the pandemic, which would not yet have occurred. Regarding PIL and MC, we could not compare our results with those of other authors because, to the best of our knowledge, this is the first study to longitudinally examine these dimensions in the development of psychopathology and burnout in HCWs during COVID-19. In fact, existing studies are on the meaning in life (which is a much broader concept) (42) or on moral distress (which is a different term than MC) (43). On the other hand, this research was carried out at a single hospital, which, together with the small sample size, may have reduced its external validity compared to multicentre studies of larger sample sizes. Although the inclusion of both clinical and non-clinical staff as HCWs may be considered a limitation, we would like to point out that non-clinical staff continued to work and have contact with patients during the COVID-19 pandemic, as did clinical staff. Therefore, we want to recognise their work during the pandemic but also acknowledge their differences from clinical staff.

## 5. Conclusion

This study showed that even though the psychopathology caused by the COVID-19 pandemic in HCWs has decreased as time has passed, its prevalence is still high. Personal and family/friends’ exposure to SARS-CoV-2 and purpose in life have been shown to be predictors of psychopathology at the beginning of the pandemic. Although purpose in life predicted the onset of psychopathology, it seems that once the psychopathology is established, the factors responsible for its maintenance will be others. For this very reason, the role of moral courage may have been overshadowed by other factors, such as purpose in life. The present research could be useful to get an idea of the evolution of the mental health of healthcare workers in future epidemics/pandemics and the importance of strengthening the purpose in life and moral courage of workers to avoid initial psychopathology and change its tendency during a health crisis. Finally, it also supports future longitudinal studies on the evolution of post-pandemic psychopathology and the role of purpose in life and moral courage on it.

## Data availability statement

The raw data supporting the conclusions of this article will be made available by the authors, without undue reservation.

## Ethics statement

The studies involving humans were approved by Investigation Commission at the Provincial Hospital Consortium in Castellon (ref. A-15/04/20) and the Clinical Research Ethics Committee at the Cardenal Herrera-CEU University (ref. CEI20/068). The studies were conducted in accordance with the local legislation and institutional requirements. The participants provided their written informed consent to participate in this study.

## Author contributions

IE: Conceptualization, Data curation, Formal analysis, Methodology, Writing – original draft, Writing – review & editing. LR-J: Data curation, Investigation, Writing – original draft, Writing – review & editing. AB: Conceptualization, Formal analysis, Methodology, Supervision, Writing – review & editing. LAR-B: Data curation, Investigation, Writing – review & editing. MO’H: Data curation, Investigation, Writing – review & editing. GH: Conceptualization, Funding acquisition, Project administration, Supervision, Writing – review & editing.
